# Characteristics and phylogenetic analysis of the complete mitochondrial genome of *Microstomus achne*

**DOI:** 10.1080/23802359.2023.2172966

**Published:** 2023-02-09

**Authors:** Jun Young Chae, JinHo Kim, Tae-Wook Kang, Jinkoo Kim, Hyung-Ho Lee, Moo-Sang Kim

**Affiliations:** a Department of Bioinformatics, the MOAGEN, Daejeon, South Korea; b Department of Biotechnology, Pukyong National University, Busan, South Korea; c Major of Marine Biology, Pukyong National University, Busan, South Korea

**Keywords:** *Microstomus achne*, mitogenome

## Abstract

*Microstomus achne* (Jordan and Starks, 1904) is an economically valuable flatfish belonging to the family Pleuronectidae and the only flatfish that inhabits Korea. Here, we report on the complete mitochondrial genome of *M. achne* and the phylogenetic relationship between close species. The mitogenome is 16,971 bp long and encodes 13 protein-coding genes (PCGs), 22 transfer RNAs, and two ribosomal RNAs. The phylogenetic analysis showed that *M. achne* clustered with *Glyptocephalus stelleri*, which supports the conclusion that *M. achne* belongs to the family Pleuronectidae. The results of this study provide a better understanding of *M. achne*.

## Introduction

*Microstomus achne* (Jordan and Starks, 1904) is a benthic fish belonging to the order Pleuronectiformes and family Pleuronectidae, and its most striking morphological characteristics is the white spots on the body (Cooper and Chapleau 1998). The genus *Microstomus* was reported to comprise 10 nominal species worldwide, five of which have recently been excluded from the genus, and four of which are currently accepted as valid (Norman 1934; Cooper and Chapleau 1998; Froese and Pauly 2022). Of this genus, only *M. achne* is known to inhabit Korea (Kim et al. 2005). However, genetic information on members of these genera is remarkably limited, and in particular, no complete mitochondrial DNA sequences have yet been reported for this genus. We describe the completed mitochondrial genome of *M. achne*, which was acquired using next-generation sequencing, and we anticipate that this information will help to understand the phylogenetic status of *M. achne*.

## Materials

**Figure 1.**
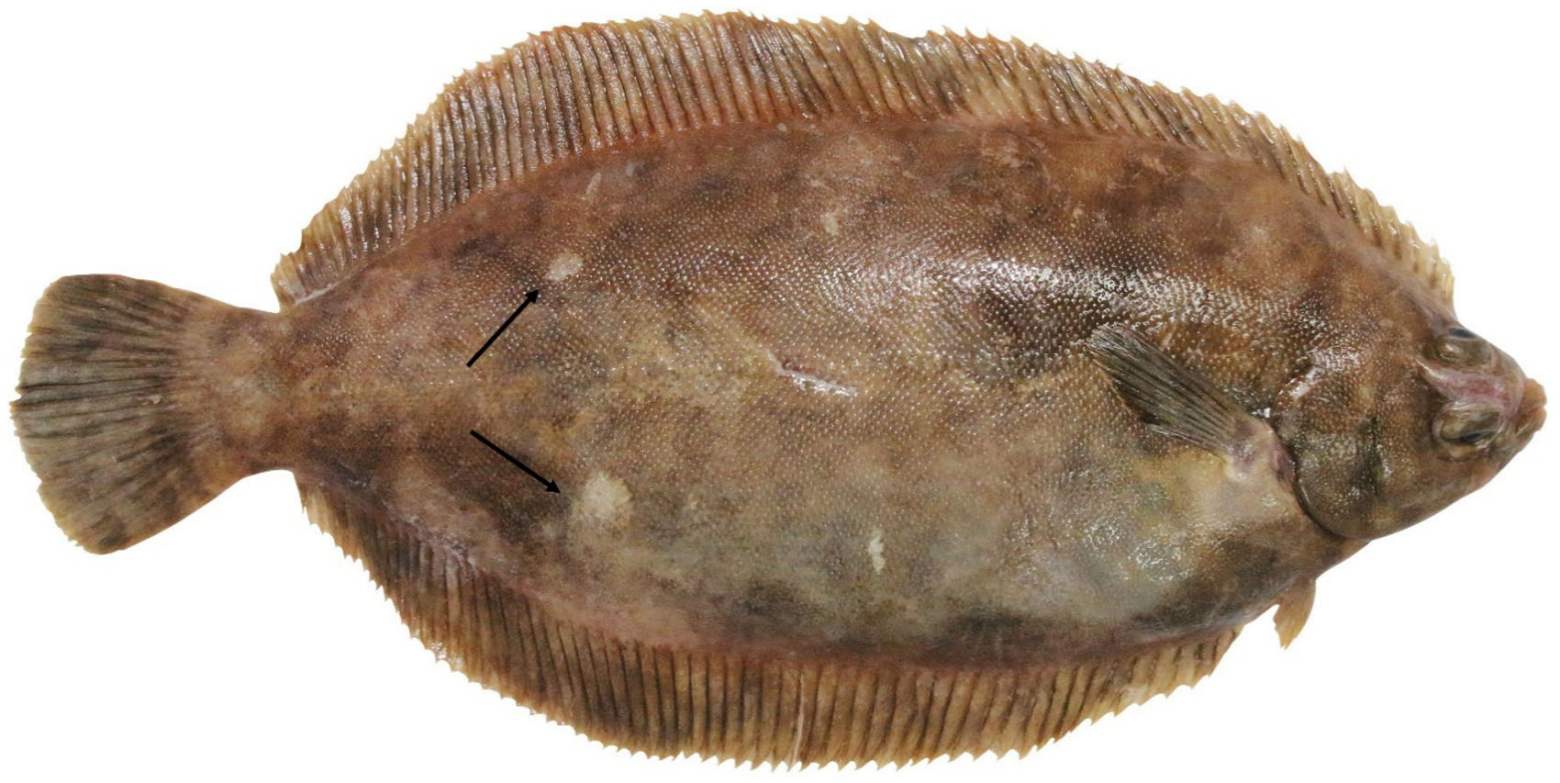
*Microstomus achne*. The sample from which a fin was utilized in this study. This photo was provided by Prof. Jinkoo Kim. The white spots on the body of *M. achne*, which can be used to identify the flatfish species, are marked with black arrows.

We obtained the fin of a flatfish fin that was collected from Boryeong South Korea (36°22′N, 126°34′E) and deposited in the Pukyong National University storage facility (Figure 1) from the Marine Fish Resource Bank of Korea under Voucher no. MRS002000076819 (https://www.mbris.kr/pub/main/publicMainPage.do; Jinkoo Kim, tjgk2002@gmail.com).

## Methods

The genomic DNA was extracted using the PureHelix™ Genomic DNA Prep Kit [Animal], Solution Type (NANOHELIX, Daejeon). For species identification using the *cox1* sequence, the *cox1* gene was amplified by PCR using the fish universal primer set (Ward et al. 2005) and sequenced by Macrogen (South Korea). The partial *cox1* sequence was compared using a BLASTN (Johnson et al. 2008) search.

Raw data from next-generation sequencing were obtained using the method described in Chae et al. (2022) and then deposited in the Sequence Read Archive (SRA) database (SRR21722070). The raw data were trimmed with Cutadapt ver. 4.1 (Martin 2011), and a contig sequence was produced with the default option in the *de novo* assembler of the CLC Genomics Workbench (ver. 20.04; QIAGEN). The circular form of the mitogenome was confirmed by using the “Map to Reference” tool in Geneious software (ver. 2021.2.2; https://www.geneious.com) to map the filtered data onto the contig sequence. Annotation of this final sequence was performed in the MITOS Webserver (Bernt et al. 2013), after which the detailed annotation was manually corrected using SnapGene software (ver. 5.3.2; GSL Biotech LLC; snapgene.com). Finally, the completed circular form of the mitogenome sequence was registered at the NCBI GenBank (OP066370). The mitogenome map was prepared using ORDRWA (Greiner et al. 2019).

The phylogenetic tree was constructed with Mr Bayes ver. 3.2.6 (Huelsenbeck and Ronquist, 2001), using mitogenomes from 20 members of the class Actinopteri, including *Acipenser fulvescens* (MT667238) as an outgroup (*Cleisthenes herzensteini*, KT223828 [Bo et al. 2016]; *Pseudopleuronectes herzensteini*, ON127848 [Chae et al. 2022]; *Cynoglossus semilaevis*, EU366230 [Kong et al. 2009]; *Arnoglossus tenuis*, KP134337 [Li et al. 2015]; *Paraplagusia blochii*, JQ349002 [Li et al. 2016]; *Pseudopleuronectes yokohamae*, KT878309 [Liu et al. 2017]; *Kareius bicoloratus*, AP002951 [Miya et al. 2001]; *Hippoglossus hippoglossus*, AM749122, *Hippoglossus stenolepis*, AM749126, *Reinhardtius hippoglossoides*, AM749130 [Mjelle et al. 2008]; *Pleuronichthys cornutus*, JQ639071 [Shi et al. 2013]; *Cynoglossus sinicus*, JQ348998 [Shi et al. 2015]; *Cynoglossus roulei*, MK574671 [Wang et al. 2020]). Each mtDNA sequence was retrieved from GenBank. These nucleotide sequences of protein-coding genes (PCGs) were aligned and analyzed using a GTR substitution model and 1,100,000 chain length.

## Results

The BLASTN search showed that our sample shared the highest identity with *M. achne* (MH032470.1) at 99.81%, suggesting that our flatfish is *M. achne*. Its sequence identity with *M. shuntovi* (MH032479.1), *Embassichthys bathybius* (MH032412.1), *M. kitt* (MN122933.1), and *M. pacificus* (MH032476.1) decreased in the order of 98.72%, 93.61%, 92.85%, and 90.68%, respectively.

**Figure 2.**
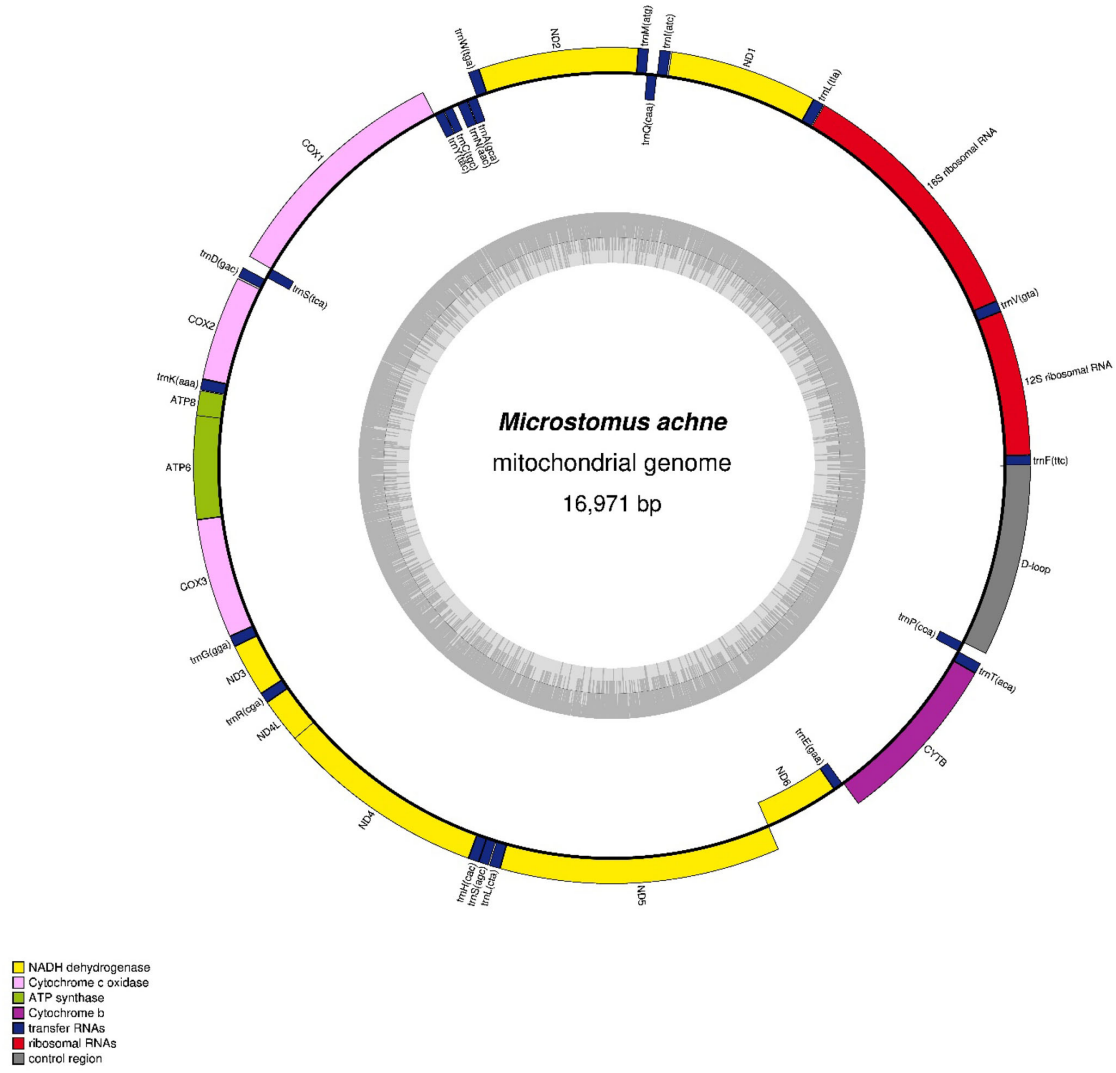
The complete mitochondrial genome map of *M. achne*. All genes in the *M. achne* mitogenome, including 13 protein-coding genes, 22 transfer RNA genes, and two ribosomal RNA genes, are represented. The GC content is represented by the inner circle.

The total length of the final mitogenome was 16,971 bp, and it comprised 13 PCGs, 22 *tRNA* genes, and two *rRNA* genes. Its gene order matched that of *Glyptocephalus stelleri* (MT258402). Among the PCGs, only *nad6* was transcribed on the negative strand, and all others were transcribed on the positive strand (Figure 2). The ATG codon was used as a start codon in 12 PCGs (*nad1*, *nad2*, *cox2*, *atp8*, *atp6*, *cox3*, *nad3*, *nad4l*, *nad4*, *nad5*, *nad6*, and *cob*), while the GTG codon was used as a start codon in *cox1. nad1*, *atp8*, *atp6*, *nad4l*, and *nad5* used the TAA stop codon. While the truncated T- codon terminated translation in *nad2*, *cox2*, *nad3*, *nad4*, and *cob*, and the truncated TA- stopped translation in *cox3*. The AGA codon and the TAG codon were utilized as stop codons in *cox1* and *nad6*, respectively.

The mitogenome of *M. achne* contained 22 *tRNA* genes, including two *tRNA-L* and two *tRNA-S*. Of these *tRNA* genes, 14 (*tRNA-F*, *tRNA-V*, *tRNA-L2*, *tRNA-I*, *tRNA-M*, *tRNA-W*, *tRNA-D*, *tRNA-K*, *tRNA-G*, *tRNA-R*, *tRNA-H*, *tRNA-S1*, *tRNA-L1*, and *tRNA-T*) were transcribed on the positive strand; the remaining *tRNA* genes (*tRNA-Q*, *tRNA-A*, *tRNA-N*, *tRNA-C*, *tRNA-Y*, *tRNA-S2*, *tRNA-E*, and *tRNA-P*) were transcribed on the negative strand (Figure 2). In the predicted secondary structure, there were standard and abnormal structures in the tRNA loop or stem. *tRNA-C* and *tRNA-S1* contained an abnormality in the D-loop. Imperfect base pairing in the T-loop was observed in *tRNA-V*, *tRNA-W*, *tRNA-M*, *tRNA-N*, and *tRNA-E*. In addition, incomplete base pairing in the acceptor stem was found in *tRNA-F*, *tRNA-V*, *tRNA-I*, *tRNA-R*, *tRNA-H*, *tRNA-L1*, and *tRNA-T*. Uniquely, *tRNA-S1* had partial base pairing in every stem. The other genes possessed standard tRNA structure.

The two *rRNA* genes were located close to the border of *tRNA-V* (Figure 2). Between *tRNA-F* and *tRNA-V*, a small *rRNA* was placed, while a large *rRNA* was placed between *tRNA-V* and *tRNA-L2* (Figure 2). The small and large *rRNA* were 951 bp and 1713 bp in length, respectively. The putative control region was located between *tRNA-P* and *tRNA-F* and had a length of 1261 bp (Figure 2).

**Figure 3.**
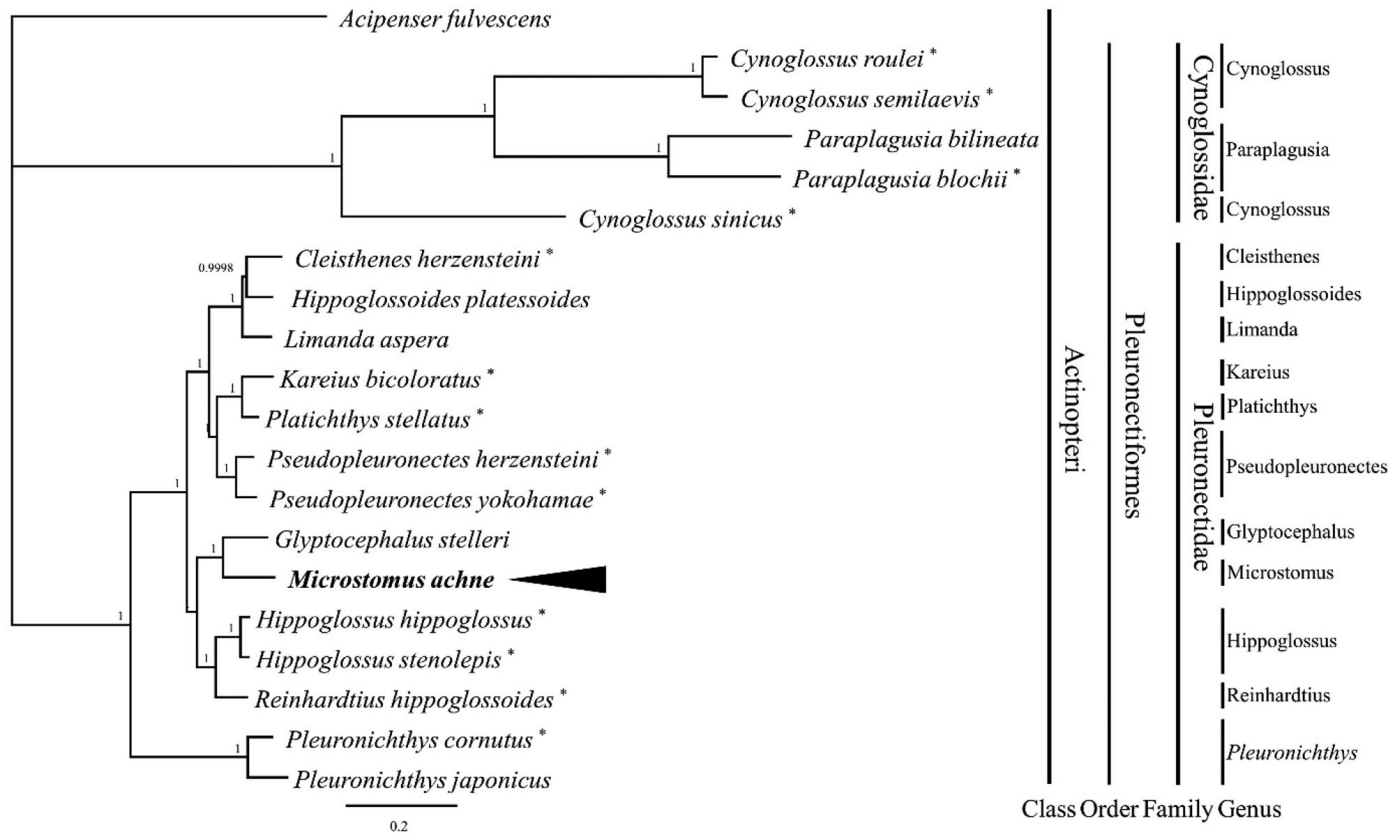
Phylogenetic tree of *M. achne* and related species. Based on Bayesian inference (BI), the phylogenetic relationship of 20 members of *Actinopteri*, including *Acipenser fulvescens* (MT667238) as an outgroup, was analyzed utilizing the nucleotide sequences of 13 mitogenomic protein-coding genes. The node numbers correspond to the Bayesian inference’s posterior probabilities. The black arrow indicates the *M. achne* analyzed in this study. The presence of a star next to a species name indicates that this mitogenome was published. The following sequences were used: *Cleisthenes herzensteini*, KT223828 [Bo et al., 2016]; *Pseudopleuronectes herzensteini*, ON127848 [Chae et al., 2022]; *Cynoglossus semilaevis*, EU366230 [Kong et al., 2009]; *Arnoglossus tenuis*, KP134337 [Li et al., 2015]; *Paraplagusia blochii*, JQ349002 [Li et al., 2016]; *Pseudopleuronectes yokohamae*, KT878309 [Liu et al., 2017]; *Kareius bicoloratus*, AP002951 [Miya et al., 2001]; *Hippoglossus hippoglossus*, AM749122, *Hippoglossus stenolepis*, AM749126, *Reinhardtius hippoglossoides*, AM749130 [Mjelle et al., 2008]; *Pleuronichthys cornutus*, JQ639071 [Shi et al., 2013]; *Cynoglossus sinicus*, JQ348998 [Shi et al., 2015]; *Cynoglossus roulei*, MK574671 [Wang et al., 2020].

Each species was identified as belonging to either family Pleuronectidae, family Cynoglossidae, or the outgroup (*A. fulvescens*). *M. achne* clustered with *G. stelleri* of the family Pleuronectidae and was separated from other nodes in the phylogenetic analysis (Figure 3).

## Discussion and conclusion

*Microstomus achne* is the only flatfish belonging to the genus *Microstomus* that can be observed in South Korea. In addition, this genus lacks any complete mitogenomes. Here, the complete mitochondrial genome of *M. achne* was identified, and its genetic characteristics were elucidated. Significantly, the gene composition was the same as the general mitogenome composition of vertebrates (Pereira, 2000). In addition, gene composition and order had no significant features compared with the family Pleuronectidae. This is the first study to report the complete mitogenome of flatfish of the genus *Microstomus*. These data can be utilized to reveal the phylogenetic relationships between members of the genus *Microstomus*, especially *M. achne*.

## Data Availability

The genome sequence data supporting this study’s findings are available in the GenBank of the NCBI at (https://www.ncbi.nlm.nih.gov/) under accession no. OP066370. The associated BioProject, SRA, and Bio-Sample numbers are PRJNA882383, SRR21722070, and SAMN30933649, respectively.
